# Is Maternity Blues a risk factor for the onset of post-partum depression? A longitudinal Study

**DOI:** 10.1192/j.eurpsy.2022.942

**Published:** 2022-09-01

**Authors:** V. Sollo, F. Zinno, A. Vece, V. Giallonardo, G. Sampogna, M. Luciano, A. Fiorillo

**Affiliations:** 1 Università della Campania “Luigi Vanvitelli”, Dipartimento Di Psichiatria, Napoli, Italy; 2 University of Campania “Luigi Vanvitelli”, Department Of Psychiatry, Naples, Italy; 3 University of Campania, Department Of Psychiatry, Naples, Italy

**Keywords:** maternity, blues, Postpartum, Depression

## Abstract

**Introduction:**

The period after delivery is characterised by physical, hormonal and psychological changes. Up to 20% of women can present depressive and anxiety symptoms and difficulties in the interaction with the newborn, emotional lability. This condition is also called “Maternity Blues (MB)”.

**Objectives:**

To: 1) assess the frequency of MB presentation of depressive symptoms immediately after the delivery; 2) identify those characteristics more frequently associated to the onset of depressive symptoms after the delivery; and 3) verify the hypothesis that the presence of maternity blues is a risk factor for the onset of a depressive episode in the 12 months after the delivery.

**Methods:**

From December 2019 to February 2021 all women who gave birth at the University of Campania “Vanvitelli” were enrolled. Upon acceptance, they filled in the EPDS Scale. Sociodemographic, gynaecological, peripartum and psychiatric anamnesis was collected at baseline. Women have been reassessed after 1, 3, 6 and 12 months.

**Results:**

359 women were recruited, with a mean EPDS score of 5.51. Among these, 83 reported the presence of MB (EPDS score≥10; 23.12%). Anxiety disorders with onset prior to pregnancy (p<.000), preeclampsia (p<.01), increased foetal health rate (p<.01), conflicts with relatives (p<.001) and anxiety disorders the partner (p<.01) emerged as predictors of Mb. The presence of MB increase 7 time the risk to have higher EPDS score at follow-up assessments (p<.000).

**Conclusions:**

The presence of MB should always be assessed in the immediate post-partum and psychosocial interventions should be provided to women with MB to reduce its potential negative effect on mental health.

**Disclosure:**

No significant relationships.

